# Fast and noise-tolerant determination of the center of rotation in tomography

**DOI:** 10.1107/S1600577521012777

**Published:** 2022-01-19

**Authors:** Everett Vacek, Chris Jacobsen

**Affiliations:** a Applied Physics Program, Northwestern University, 2145 Sheridan Road, Evanston, IL 60208, USA; b Advanced Photon Source, Argonne National Laboratory, 9700 South Cass Avenue, Argonne, IL 60439, USA; c Department of Physics and Astronomy, Northwestern University, 2145 Sheridan Road, Evanston, IL 60208, USA; d Chemistry of Life Processes Institute, Northwestern University, 2170 Campus Drive, Evanston, IL 60208, USA

**Keywords:** X-ray tomography, center of rotation, tomogram alignment

## Abstract

High-quality tomographic reconstruction is not possible without the accurate localization of the center of rotation. Poor localization leads to artifacts in the data and can even cause reconstructions to fail. There are many approaches to solving this problem, some of which involve the collection of full sinograms, or even provisional tomographic reconstructions, in order to determine the center of rotation. Here, a simple method based on the expected symmetry of the Fourier transform of summed projections approximately 180° apart is presented; unlike cross-correlation methods, it requires only a single Fourier transform to compute, and uses mainly low spatial frequency information which is less susceptible to noise. This approach is shown to be fast, and robust against poor signal-to-noise as well as to projection images acquired at angles that are not exactly 180° apart. This rapid method can be useful as a first step in the processing of tomographic data.

## Abstract 2

A rapid phase symmetry method for finding the center of rotation in tomographic data using two images located 180° ± Δθ apart is introduced. The method is more robust against photon noise than image correlation methods, and shows good tolerance to Δθ ranging up to several degrees.

## 1. Introduction

**Figure 1:**
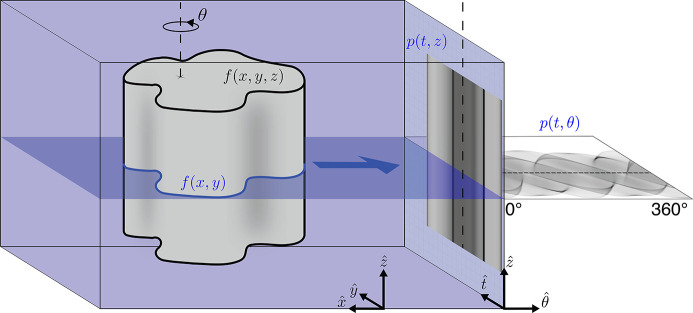
Parallel beam tomographic imaging of an object *f*(*x*, *y*, *z*) with a rotation axis in the 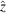 direction. X-rays moving from left to right pass through the object producing a projection image *p*(*t*, *z*) on the detector (grid on far right). The object is rotated and imaged iteratively, producing the full tomographic data set *p*(*t*, θ, *z*). The vertical dashed lines indicate the location of the axis of rotation in each of the three domains. If the axis of rotation is aligned with the 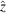 axis, we can reconstruct 2D slices of the object *f*(*x*, *y*) at *z* from sinograms *p*(*t*, θ) at *z*.

In single-tilt-axis projection tomography, a set of 2D slices of the object are reconstructed, with each slice arising from a set of line projections obtained as the object is rotated as shown in Fig. 1 ▸. [Some electron microscopes have double-tilt-axis specimen stages (Penczek *et al.*, 1995 ▸); hence our use of the term ‘single-tilt-axis’ tomography which describes most tomography experiments at synchrotron light source facilites, and most commercial X-ray microtomography systems.] However, if one uses an incorrect value for the position of the actual axis of rotation, objects appear to ‘wobble’ about the correct axis, resulting in ‘tuning fork’ artifacts in the reconstructed slice if projections are acquired over 180° (Shepp *et al.*, 1979 ▸), or one type of ring artifact for 360° data. A number of approaches have been developed to find and correct for this center-of-rotation (COR) shift error:

(i) One approach is to compare a 0° image with the mirrored version of an image taken 180° apart, and find the COR shift to be half the distance between the phase correlation offset between these two images (Gullberg *et al.*, 1986 ▸; Donath *et al.*, 2006 ▸). This operation requires a total of three Fourier transforms, as well as identification of the cross-correlation peak. Alternatively, one can use the shift of the center of mass between these two images for the same purpose (Donath *et al.*, 2006 ▸; Dong *et al.*, 2013 ▸).

(ii) One simple approach is to fit a sine function to the sinogram’s angle-by-angle center of mass and use this to calculate the COR shift (Hogan *et al.*, 1993 ▸). This approach can also be used on the raw sinogram data (Azevedo *et al.*, 1990 ▸; Donath *et al.*, 2006 ▸). One can also track the center crossing points of identified features in the sinogram (Li *et al.*, 2010 ▸). These carry the added benefit that, for low noise data sets, individual projections can be shifted such that features follow a sinusoidal path. When taking the Fourier transform of the sinogram, COR errors lead to the presence of aliasing errors at high spatial frequencies (Edholm *et al.*, 1986 ▸), which can be minimized in an alignment strategy (Vo *et al.*, 2014 ▸).

(iii) Because of the effects on the reconstructed slice image, one can carry out reconstructions with a range of choices for the COR shift and manually select the one with the best appearance (Walls *et al.*, 2005 ▸). Automated approaches include maximizing the number of positive, non-zero pixels in the reconstructed slice (Brunetti & De Carlo, 2004 ▸), or the contrast of the slice image (Birk *et al.*, 2010 ▸), or minimizing the image’s total variation (Cheng *et al.*, 2018 ▸). One can also train a neural network to recognize ring-like artifacts and use this network to identify the image with the correct COR shift (Yang *et al.*, 2017 ▸).

(iv) In an iterative reprojection approach, one first obtains a provisional 3D image and then aligns projection images to this volume, repeating until convergence is achieved (Dengler, 1989 ▸; Owen & Landis, 1996 ▸; Birk *et al.*, 2010 ▸; Parkinson *et al.*, 2012 ▸). This approach can also be applied in reciprocal space (Pryor *et al.*, 2017 ▸), and to a single slice in the reconstructed object. Iterative reprojection can correct for additional errors beyond the COR shift, and speedups can be obtained by alternating between alignment steps and reconstruction steps in iterative reconstruction approaches (Gürsoy *et al.*, 2017 ▸).

Of these approaches, the first involves minimal data and computation. The second requires full sinogram data, and somewhat longer computation time. The third and fourth approaches require numerous reconstructions, either of a single slice with varying COR guesses (the third approach) or of the entire 3D data set (the fourth approach) with consequent costs in data storage and compute time.

We present here a rapid approach for determination of the COR shift based on phase symmetry in the frequency domain of two projection images obtained approximately 180° apart. This resembles the first approach listed above with some distinct differences. First, while phase correlation finds the shift between a projection and the mirror of its 180° counterpart, it normally involves three two-dimensional Fourier transforms, whereas the phase symmetry approach requires only a single one-dimensional Fourier transform. Second, we make use of the fact that this phase symmetry is quite insensitive to noise at low spatial frequencies of the projection images, as well as being insensitive as to whether the images were taken exactly 180° apart. This rapid COR shift determination method can be used alone, or to provide a highly accurate starting point for correction of additional errors in the third and fourth approaches described above.

## 2. Method

**Figure 2:**
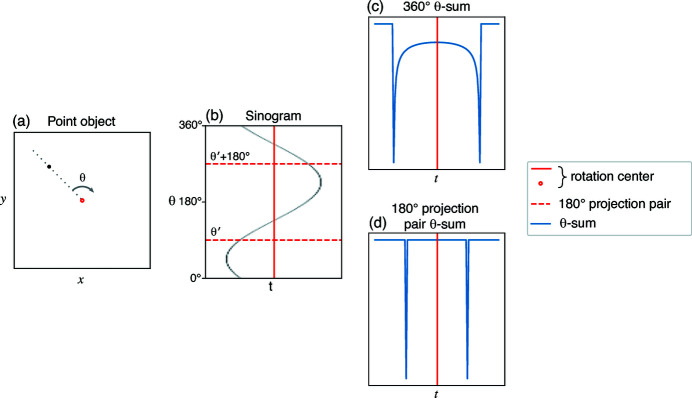
Phase symmetry of a point in motion about an axis of rotation (*a*). A point object gives a simple sinusoid in the sinogram (*b*). The θ-sum of the full 360° sinogram gives an arcsine distribution symmectric about the rotation axis (*c*). Since any 180° projection pairs sample points on opposite ends of the θ-sum, they retain the same symmetry (*d*).

For an object rotated along the 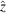 axis as shown in Fig. 1 ▸, single point features will produce sinusoids in the sinogram *p*(*t*, θ) (using the notation from Fig. 1 ▸); therefore 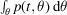 for a single feature will result in an arcsine distribution for emission tomography and an inverted arcsine distribution in the case of transmission tomography. This distribution has even symmetry about the rotation axis as shown in Fig. 2 ▸(*c*). Since we collect a discrete number of projections, the integral becomes a sum over those projections, 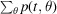 , which we will call the θ-sum of a sinogram. We may also consider a reflection pair, which we define as the sum of one projection taken at θ′ and another taken at θ′ + 180°. For single point features, these projection pairs will sample portions of the arcsine distribution on opposite ends of the rotation axis, resulting in the same even symmetry as shown in Fig. 2 ▸(*d*).

**Figure 3:**
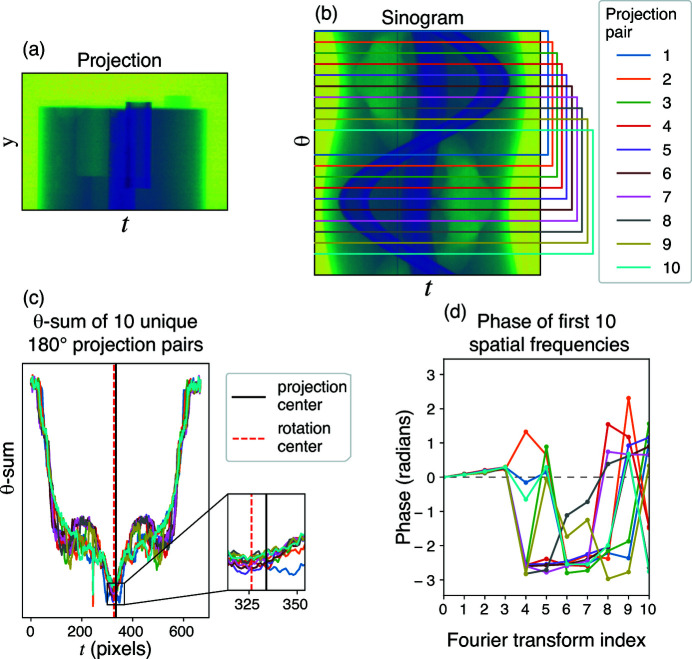
Demonstration of the projection pair approach. Using data set tomo_00030 from *TomoBank* (De Carlo *et al.*, 2018 ▸) (*a*), ten sets of θ-sums were obtained from projection pairs that were each separated by 180° in the sinogram (*b*). The corresponding θ-sums for each projection pair are shown in (*c*), with both the projection center as recorded in the data set and the recovered rotation center shown. Because an offset from centrosymmetry leads to a phase ramp in Fourier space, Fourier transforms of each of these θ-sums shown in (*d*) all show the same phase ramp at the first spatial frequency index (that is, the lowest spatial frequency in the Fourier transform), with higher spatial frequencies then reflecting variations due to what exact features are present in the projection pair. The correct rotation center is recovered from the phase of the first spatial frequency index.

A continuous object can be thought of as an ensemble of single point features. This will result in a linear combination of arcsine distributions in the θ-sum which will necessarily retain the same symmetry about the rotation axis. If, however, the actual rotation axis is shifted from the center of the recorded projection due to a COR error (as shown in Fig. 3 ▸), the distribution will be offset; one will instead have a non-centrosymmetric distribution.

A simple way to determine whether a function is non-centrosymmetric is to examine its Fourier transform, since the shift theorem relates an offset center in real space to a phase ramp in Fourier space. If one did this row by row in the reflection pair, one could separate out erroneous contributions to the phase ramp from rows with no features (but only noise), or rows with other types of errors. When taking the Fourier transform of the sum of all rows in the reflection sum, these erroneous contributions would contribute equally to the phase ramp, whereas, if one were to take the Fourier transform of individual rows one by one and then obtain the summation of these complex vectors, the non-erroneous rows would contribute to the phase ramp in a correlated fashion while the noise- or error-corrupted rows would presumably contribute in a non-correlated fashion. Therefore for *N*_*t*_ pixels across the detector for each of *N*_*y*_ detector rows, one is best off either adding the contributions of a total of *N*_*y*_ Fourier transforms each of dimension *N*_*t*_, or a single Fourier transform of the (*N*_*t*_, *N*_*y*_) data array rearranged as a 1D array of dimension *N*′ = *N*_*t*_ *N*_*y*_; we chose to use the latter approach.

**Figure 4:**
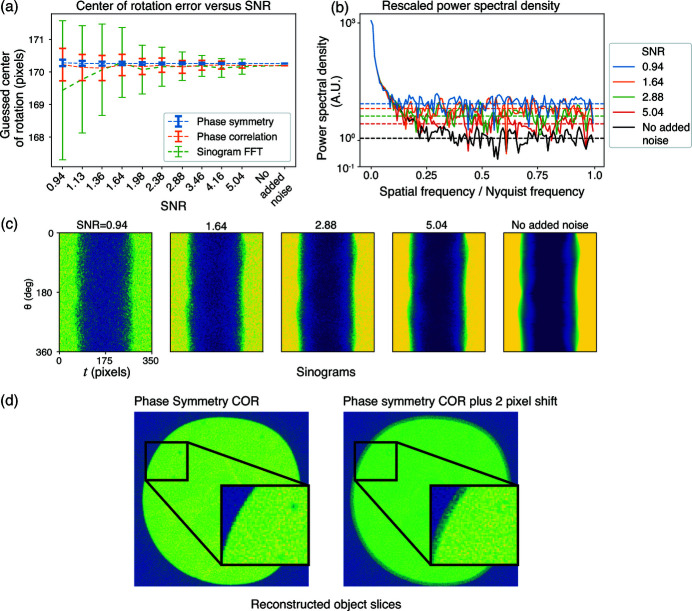
The effect of varying SNR on algorithms for finding the center of rotation (COR) for the tomo_00064 data set. In (*a*), we show the COR along the 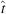 -axis as determined by three methods (phase symmetry, phase correlation, and sinogram FFT) for real data with simulated noise. Center finding was repeated for 100 instances of simulated Poisson noise for each value of 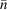 and resulting SNR as summarized in Table 1 ▸. From these 100 instances, the mean value of the center of rotation, as well as the standard deviation of the results, are shown for each SNR value. In (*b*), the power spectral density for the reflection image pair is shown for a smaller subset of SNR values, with all curves normalized to the same power at zero spatial frequency; as expected from Fig. 3 ▸(*d*), the lowest spatial frequencies are least affected by noise. The sinograms versus SNR are shown in (*c*). In (*d*), a reconstruction of the sinogram is done for the COR as determined by the phase symmetry method (left), and also for a 2 pixel offset in the COR (right); as can be seen, the image on the left shows no ring or tuning-fork artifacts, while the image on the right shows ring artifacts as expected for this 360° data set.

The fast Fourier transform of this *N*′ = *N*_*t*_ *N*_*y*_ reflection sum array will contain information about the phase ramp resulting from a COR error. However, the magnitudes in the Fourier transforms of images tend to decline as a function of increasing spatial frequency (Burton & Moorhead, 1987 ▸; van der Schaaf & van Hateren, 1996 ▸), while uncorrelated noise due to photon statistics is equally present at all spatial frequencies. In addition, the appearance of different features in different instances of projection pairs will affect higher spatial frequencies in the θ-sum. Therefore one will have the highest fidelity measurement of the presence of any phase ramp at the lowest non-zero spatial frequency *f*_min_ in the FFT of the *N*′ reflection sum array, as shown in Fig. 3 ▸(*d*) and in Fig. 4 ▸(*b*) below. In principal we could improve the speed of this method by calculating this lowest spatial frequency value only, such as by using the Goertzel algorithm (Goertzel, 1958 ▸) with only *N*′ multiplications and 2*N*′ additions. However, the optimizations coded into standard FFT routines [which require 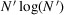 operations] make the full 1D FFT calculation quite fast enough. Thus we calculate the phase ramp ϕ(*f*_min_) from the real *R* and imaginary *I* parts of the lowest spatial frequency of the FFT of the projection pair θ-sum as 

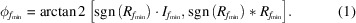

where 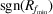 is the sign of the real component, which accounts for transmission or emission tomography. We convert the phase shift 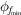 to a position shift *t*′ away from the center of our sinogram (the COR error) using 

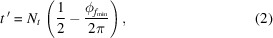

thus yielding a rapid approach to finding the COR error *t*′.

Some practical considerations to include are that the method requires the object to be totally within the frame of the image for all exposures. Otherwise, the entire arcsine distribution is not collected and the center finding will begin to fail. Also since we are using only the lowest spatial frequency the method is strongly effected by illumination gradients along the *t*-axis. More often than not, normalization to the flat- and dark-field projections corrects for illumination gradients, a standard step in any tomographic reconstruction. Further improvement can be had by normalizing the background visible on the right and left sides of the projection images to be equal. These techniques are both accessible in the *TomoPy* Python package.

## 3. Demonstration

We compared the above phase symmetry method with two other popular center-finding algorithms: phase correlation between the reflection pair of projections at θ′ and θ′ + 180°, and the sinogram FFT method which involves taking the Fourier transform of a complete sinogram. We tested the phase symmetry and phase correlation methods as a function of departures Δθ from a perfect projection pair at angles of θ′ and θ′ + 180° + Δθ. We also compared all three approaches against the signal-to-noise ratio (SNR) of the acquired projection images.

We compared these approaches by using a data set from *TomoBank* (De Carlo *et al.*, 2018 ▸) of phase contrast tomography of duplex stainless steel taken at the European Synchrotron Radiation Facility (ESRF; *TomoBank* data set tomo_00064). This data set consists of 450 projections acquired over a 360° range, with a high density of features such that one cannot easily see individual features in the sinogram. Because this data set has very high SNR, we generated a version of this data set where the sinogram was first normalized to its maximum value, then multiplied by an assumed fluence of 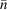 photons per pixel, and each projection image had Poisson noise added according to each pixel’s assumed photon count. One can then calculate the projection data SNR from the correlation of two separate images *I*_1_ and *I*_2_ with two independent instances of Poisson noise (Bershad & Rockmore, 1974 ▸; Huang *et al.*, 2009 ▸). Because the SNR depends on the contrast of the object, and because of statistical fluctuations due to specific instances of Poisson noise, we show in Table 1 ▸ the values of 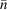 used and the resulting SNR in various tests.

### 3.1. Phase correlation method

The phase correlation method performs phase correlation in the Fourier domain on two projections separated by 180° (Gullberg *et al.*, 1986 ▸; Donath *et al.*, 2006 ▸). Since the object has been rotated 180°, the second projection image is mirrored about the 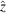 axis before processing. The cross-correlation between the two images will then give a peak at a position that is offset from the array center by twice the COR error *t*′ along the 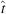 axis (it will also provide a measure of sample drift along the 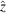 axis during data acquisition). One can further increase the accuracy of this method by correlating only on the phase of the two Fourier transforms (Shaw *et al.*, 1989 ▸). Sub-pixel accuracy can be obtained by embedding the product of the Fourier transforms of the two images in a larger array before inverse FFT, either based on the entire array or on a sub-array (Guizar-Sicairos *et al.*, 2008 ▸; van der Walt *et al.*, 2014 ▸). In our case we used a sub-pixel accuracy of a tenth of a pixel (*i.e.* *m* = 10). Conventional sub-pixel phase correlation would require the *N*_*t*_ × *N*_*y*_ array be embedded in an *mN*_*t*_ × *mN*_*y*_ array before inverse FFT. A less memory intensive approach obtains a coarse estimate of the correlation peak on an *N*_*t*_ × *N*_*y*_, then obtains a sub-pixel estimate of the correlation peak using matrix-multiplication discrete Fourier transform (DFT) only on a sub-array around the coarse estimate. Thus while the phase symmetry approach requires only a single 1D FFT of length *N*_*t*_ *N*_*y*_, the phase correlation method requires either two *N*_*t*_ × *N*_*y*_ FFTs and one *mN*_*t*_ × *mN*_*y*_ inverse FFT (for the conventional approach) or two *N*_*t*_ × *N*_*y*_ FFTs, one *N*_*t*_ *N*_*y*_ inverse FFT, and one matrix-multiplication DFT on a small array.

### 3.2. Sinogram FFT method

When one has a complete 360° sinogram over a wide range of projection angles and no COR error, its Fourier transform should only have significant values inside a double wedge region of the transform (Edholm *et al.*, 1986 ▸). Therefore the sinogram FFT method (Vo *et al.*, 2014 ▸) finds the COR error by taking a 180° sinogram and mirroring it about the 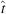 axis so as to form a 360° sinogram prior to taking its FFT. By shifting the latter 180° of the sinogram along the 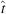 -axis, one can find where the signal outside of the double wedge is lowest, and thus recover the COR error. Since this sinogram FFT method requires repeated sinogram shifting and FFT calculation over a range of test values for the COR error, it tends to be slower than both the phase symmetry and phase correlation methods.

### 3.3. Test versus projection data SNR

The phase symmetry method is able to determine the COR error from using the overall phase ramp in the Fourier transform of a reflection pair, which can be measured from the lowest non-zero spatial frequency. As noted in Section 2, because signal in the FFT tends to decline with spatial frequency while the effects of Poisson noise do not, we first tested reproducibility of the three methods in the presence of noise while also examining the power spectrum of the sinogram pair FFT. The results given in Fig. 4 ▸(*a*) show that the phase symmetry method is the most robust of the three tested approaches against low SNR. The reason is made clear in Fig. 4 ▸(*b*), which shows the power spectrum from the FFT of the reflection pair of projections used in the phase symmetry and phase correlation methods. In this test, the phase symmetry method calculation was 32 times faster than phase correlation, and 640 times faster than the sinogram FFT approach.

### 3.4. Test against non-180° projection pairs

The phase symmetry and phase correlation methods require only two projections acquired exactly 180° apart, which we term a ‘reflection pair’. To examine the robustness of these approaches, we tested them against angular departures Δθ between this reflection pair relationship; that is, we tested the methods using one projection at θ′ against a second projection at θ′ + 180° + Δθ. These rotation errors can occur for multiple reasons:

(i) Pure projections separated by exactly 180° carry redundant information, so they are not always collected. One might instead collect *N*_θ_ rotations over an angular range of 180(1 − 1/*N*_θ_)°.

(ii) Some non-sequential projection acquisition schemes do not inherently collect 180° projections (Köhler, 2004 ▸; Münch, 2011 ▸).

(iii) Angular errors in rotation stages can result in un­intentional deviations from 180°.

**Figure 5:**
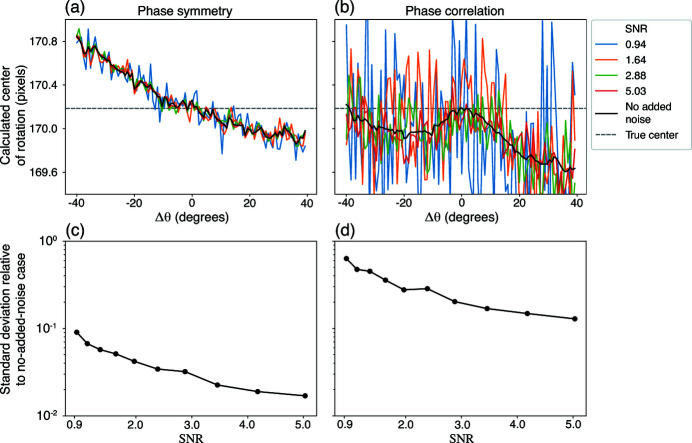
Center of rotation error (COR) recovered using the phase symmetry and phase correlation methods when using a relection pair of images obtained at rotational angles that are not exactly 180° apart: that is, with at angles θ′ and θ′ + 180° + Δθ. At each instance of SNR (Table 1 ▸), 100 instances of Poisson noise were generated. At the top we see the mean values for the recovered COR values from the phase symmetry (*a*) and phase correlation (*b*), while in the bottom row we see the standard deviation of the recovered COR values from the phase symmetry (*c*) and phase correlation (*d*) methods. Both methods deviate away from the true center as Δθ gets further from 0°, but with slightly less error and considerably lower fluctuations when using the phase symmetry approach.

Therefore, for each value of Δθ and of SNR (Table 1 ▸), we generated 100 projection pairs with different instances of simulated Poisson noise, and averaged the COR error determined using each method. The results shown in Fig. 5 ▸ indicate that the phase symmetry approach outperforms phase correlation. This is true both for producing more accurate results even with relatively large values of Δθ of ±30°, and also at lower SNR values.

### 3.5. Dose fractionation in full-rotation data sets

Dose fractionation states that the total number of photons required to generate a 3D tomographic reconstruction is the same as the number of photons required to produce a 2D projection image of a given sample at the same SNR; that is, one can divide the required dose among *N*_θ_ projection angles (Hegerl & Hoppe, 1976 ▸). Success in tomographic dose fractionation requires the alignment of low-dose projections (McEwen *et al.*, 1995 ▸). We have previously shown that the phase correlation method using differential phase contrast images provides good alignment for both transmission and X-ray fluorescence tomographic data sets (Hong *et al.*, 2014 ▸). Given that Figs. 4 ▸ and 5 ▸ show that the phase symmetry method works well at low SNR, we also tested its ability to accommodate dose fractionation.

**Figure 6:**
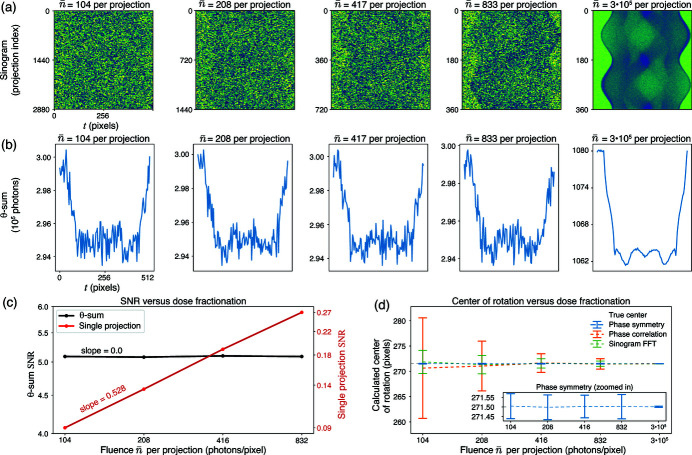
Center of rotation (COR) demonstration on dose fractionated, 360°, simulated data. A simulated low-contrast 3D Shepp–Logan phantom was generated to which Poisson noise was applied with a cumulative fluence of 300000 photons per pixel. As this fluence is divided into *N*_θ_ = 360, 720, 1440, and 2880 projections (with 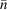 = 833, 417, 208, and 104 photons per pixel per projection, respectively), one can see that individual projections become increasingly noisy in a subset of projections shown in a sinogram (*a*) and also in the single projection SNR (*c*). Since the same cumulative fluence was used, the θ-sums shown in (*b*) and the θ-sum SNR shown in (*c*) are the same no matter the number of projections *N*_θ_ used. [A sinogram with 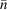 = 300000 photons per pixel per projection is also shown on the right in (*a*) and (*b*), showing no visible Poisson noise.] In spite of the very low SNR of individual projections (which scales as 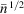 as expected) shown in (*c*), the phase symmetry method accurately finds the center of rotation with low variance as shown in (*d*). The COR error bars in (*d*) represent the standard deviation in COR results from 100 different instances of Poisson noise at the indicated fluence per projection.

To test this, we generated sinograms from a 3D Shepp–Logan phantom (Shepp & Logan, 1974 ▸) available in *TomoPy* (Gürsoy *et al.*, 2014 ▸). With this 512^3^ voxel data set, we would expect to require *N*_θ_ = 512(π/2) = 800 rotation angles to fulfill the Crowther criterion (Crowther *et al.*, 1970 ▸) for full sampling of the 3D volume. The created object had a median transmission of 0.989, so that detecting this change in transmission relative to the incident beam would suggest an object contrast parameter Θ = |*I*_max_ − *I*_min_|/(*I*_max_ + *I*_min_)^1/2^ of Θ = 0.0078, leading to an expectation that the cumulative incident fluence should be about 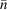 = 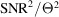 = 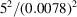 = 410000 photons per pixel. Using a slightly lower cumulative fluence of 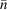 = 300000 photons per pixel for a somewhat more challenging test, we distributed this cumulative fluence over *N*_θ_ = 360, 720, 1440, and 2880 angles as one would do if they were collecting a dose-fractionated data set (yielding a fluence per projection of 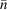 = 833, 417, 208, and 104 photons per pixel, respectively). The resulting sinograms shown in Fig. 6 ▸(*a*) illustrate the low SNR of the individual projection images at low fluence, with the per-projection SNR shown in Fig. 6 ▸(*c*). The θ-sum plots in Fig. 6 ▸(*b*) and the θ-sum SNR shown in Fig. 6 ▸(*c*) show that the cumulative fluence of 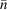 = 300000 is identical for all the dose-fractionated data sets. As shown in Fig. 6 ▸(*d*), the phase symmetry method finds the center of rotation (COR) from a reflection pair of projections quite well when dose fractionation is employed, even for the *N*_θ_ = 2880 case of per-projection fluence of 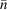 = 360 and per-projection SNR = 0.09, while the phase correlation and sinogram FFT methods perform less well.

### 3.6. Comparison testing with additional data sets

In order to make sure that the phase symmetry method can be more broadly applied, we examined a number of additional data sets. Since *TomoBank* (De Carlo *et al.*, 2018 ▸) has only a few 360° data sets but several 180° data sets, we chose two projections not 180° apart but instead two that were 179° apart, such as 0° and 179° in one test and then 1° and 180° in a second test. As shown in Table 2 ▸, near-consistency in the center of rotation calculated for these two independent (near) reflection pairs of projections indicates repeatability of the approach.

## 4. Conclusion

Phase symmetry provides a way to find the center of rotation (COR) error *t*′ of a tomographic data set in a rapid and noise-tolerant fashion. By adding together a reflection pair of projection images (two projections acquired approximately 180° apart, with some tolerance for departures Δθ from exactly 180° as shown in Fig. 5 ▸) and examination of the lowest non-zero spatial frequency of their Fourier transform, one can obtain the COR error *t*′ using equation (2). Because signal from the object tends to be much larger than Poisson noise at low spatial frequencies in Fourier transforms of images, the phase symmetry approach is robust at low exposure, which is an important consideration for dose fractionation as demonstrated in Fig. 6 ▸. However, the method does require that the object is within the field of view at all projection angles so that the reflection pair arcsine distribution is not obscured, and it also assumes that the rotation axis is aligned to the axis of the 2D detector used to acquire projection images. Lastly, it is important to note that the method is sensitive to illumination gradients in the 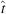 axis direction, though such gradients are normally removed as part of illumination normalization and background normalization.

### 4.1. Code

The Python code for this method can be found at https://github.com/everettvacek/PhaseSymmetry.

## Figures and Tables

**Table 1:** Assumed values of fluence 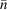 in photons per pixel used in the tests of Figs. 4 ▸ and 5 ▸, along with the resulting values of the signal-to-noise ratio (SNR) in projection images Because of the fluctuations due to different instances of estimated Poisson noise, the SNR values show slight variations between the two tests.

Fluence 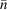	SNR (Fig. 4 ▸)	SNR (Fig. 5 ▸)
39	0.94	0.94
58	1.13	1.13
84	1.36	1.36
122	1.64	1.64
177	1.98	1.98
258	2.38	2.39
375	2.88	2.88
545	3.46	3.46
791	4.16	4.17
1150	5.04	5.03

**Table 2:** COR results on *TomoBank* (De Carlo *et al.*, 2018 ▸) data sets compared with their reported COR position in pixels, showing reproducibility of both the phase correlation approach (PC) and the phase symmetry (PS) approach described here (which offers faster calculation speed, and improved robustness against noise) Since most data sets in *TomoBank* have a total angular range of only 180°, we compared the COR calculated using two independent (near) reflection pairs: one at 0° and 179°, and another at 1° and 180°. As can be seen, the COR position is consistent to a fraction of a pixel in all cases when using the PS approach, in most other cases the PC approach gives similar values to both the PC approach and the COR position recorded in the *TomoBank* deposition (final column), with slight discrepancies in the first three data sets.

Data set	PS: 0°, 179°	PS: 1°, 180°	PC: 0°, 179°	PC: 1°, 180°	*TomoBank*
tomo_00025	950.67	950.70	952.42	952.42	952
tomo_00026	952.80	952.86	954.96	954.97	957
tomo_00031	485.84	485.89	483.82	482.14	484.5
tomo_00058	1427.06	1426.84	1426.89	1426.93	1427
tomo_00059	1440.10	1439.90	1440.38	1440.42	1440
tomo_00060	1336.71	1336.51	1336.92	1336.95	1337
tomo_00061	1315.88	1314.71	1316.27	1316.31	1316.5
tomo_00062	1359.63	1358.41	1359.90	1359.76	1359.5
tomo_00063	1321.30	1321.66	1322.02	1321.92	1322.5
